# Diphtheria—Its Diagnosis and Treatment*Read at Seguin Meeting Guadaloupe County Medical Society, October 6, 1903.

**Published:** 1903-12

**Authors:** M. B. Grace

**Affiliations:** Seguin, Texas


For Texas Medical Journal.

### Diphtheria—Its Diagnosis and Treatment.*

BY M. B. GRACE, M. D., SEGUIN, TEXAS.

In my remarks today it shall be my endeavor to present only a few practical points that will be of benefit in our actual bedside work, and not try to cover the subject that has been so often and ably presented from time to time in the different medical associations, journals and text-books.

As we are now meeting quite a number of cases, and as an epidemic is to be expected unless radical prophylactic measures are adopted, it becomes a question of immediate importance.

The question of diagnosis is where so many practitioners of today differ, hence the importance of thorough study in differentiation, for nearly all agree as regards treatment. We are called to see a child with "sore throat." Indisposition begins with a slight chilly sensation following a day or two of lassitude and followed by a temperature of any degree from 101° to 105°. Inspection reveals swollen cervical glands, closed nasal passages and mouth breathing. Tonsils and faucial pillars covered with a membrane in color anywhere from a bright, glistening, to a dirty-gray or grayish-yellow, and not easily removed with swab. We immediately state to the family that the child has diphtheria. Isolate it and

---

*Read at Seguin Meeting Guadaloupe County Medical Society, October 6, 1903.

place on proper treatment. To the children who have been exposed to this case, we administer antitoxin in from 300 to 500 units, order a bath and change of clothing for them and instruct regarding the use of simple antiseptic gargle and nasal wash, and usually feel easy over the termination. We expect recovery for our patient and continued good health for those exposed.

This is the easy side of it, but how about the difficult?

A child brought to the office, perhaps, after the day's school exercises have closed; complains of slight sore throat; temperature more than likely normal, or perhaps 99½; some degree of lassitude, but not enough to interfere with his interest in games with the other children. Inspection of the fauces discloses a small white patch on one or both tonsils. If on both, the exudate will perhaps be easily removed from one while it comes away from the other with considerable difficulty, and leaves a bleeding surface, which we may conclude too readily was due only to the manipulation.

What have we? The diagnosis is important, because the lives of other children as well as this, and the reputation of the physician depend on the accuracy of his diagnosis. Suppose he states to the family that it is a case of follicular tonsilitis, prescribes a purgative and an antiseptic throat wash, and *fails* to properly caution the people regarding isolation. The child, after two to four days, recovers without ever taking its bed. He feels secure in his diagnosis, and all is serene if it ends there. But, gentlemen, how many such cases do not end there? The physician may be called a few nights later to find his patient in the throes of a laryngeal diphtheria that soon ends fatally unless prompt and heroic measures are taken, and sometimes with them. Or, perhaps another child in the same family has a serious case of diphtheria and contracts it from the original case of so-called tonsilitis. Would it not have been better in this and all similar cases to state to the family that the diagnosis is not quite clear, and diphtheria often begins in this way, and that all such cases are to be regarded with grave suspicion until they have proven themselves? Neither with nor without a bacteriological examination can one state positively that it is not diphtheria. Frequently, early in the disease, we fail to find the Klebs-Lœfler bacillus, when a few days later the culture shows them unmistakably. Again, the facilities for making these cultures are not always at hand. The sterile culture media must be at hand. Its temperature must be kept at near the normal body heat for from eight to twenty-four hours, and after surmounting these obstacles an expert bacteriologist is sometimes essential in differentiating between the Klebs-Lœfler and the so-called pseudo bacillus.

Again, we are called to the bedside of a child with what closely resembles spasmodic croup. It will not do in such cases to simply prescribe the usual remedies and let it go at that, even though a careful inspection shows the fauces to be clean. The case should be closely watched, and upon the failure of the usual remedies, the diphtheritic nature of the trouble should be suspected and heroically treated. It is my habit to treat all suspicious laryngeal cases with antitoxin at once, not waiting for a verified diagnosis. There is another class of cases occasionally met to which I desire to call your attention. That is where the disease primarily attacks the post nasal mucous membrane. Inspection shows nothing but the slightly inflamed pharynx and fauces and perhaps some swelling of the latter. The mirror will sometimes disclose the condition behind and above the soft palate, but in case of much swelling or a rebellious child this is extremely difficult. Nasal breathing is difficult, if possible at all. After the first or second day the diagnosis is rendered easy by the fœtid breath and the nasal discharge, which varies in color from light to a dark brown or red, dependent on the quantity of blood mixed with discharge. The temperature is not a characteristic symptom in any case. It may range from 97° in one case to 105° at the beginning of another, but usually drops after the first thirty-six hours, in the latter class to 100° or 101°. The circulation does not assist in the diagnosis except when the heart beat is extremely rapid and weak. Anders, in table of differentiation, says: "In tonsilitis the deposit is soft, pultaceous, yellowish white spots or patches situated over the mouth of the follicles, with areas of redness intervening and exudation easily removed, leaving a smooth surface. In diphtheria a tough ashen gray continuous and uniform pseudo membranous deposit covers the tonsils and can be torn off in strips only, leaving a bleeding erosion."

In differentiating from the so-called pseudo diphtheria produced by the streptococcus we can only depend on the microscope, but I believe these cases extremely rare, except when associated with scarlatina or erysipelas.

The term membranous croup should be relegated to the past, where it belongs, and laryngeal diphtheria substituted, as it is not misleading.

The treatment is antitoxin, and when used in sufficient dosage in the early stages of the disease we need not fear results. In dosage each case is a law unto itself. In no class of cases will the judgment of the physician be so apparent for good as in his selection of the dose of antitoxin. The point to be held in view is to give enough. After the administration if results are not forthcoming in from twelve to eighteen hours, it may be taken as a positive indication that the dosage was not sufficient. In a case of moderate severity in a child of 2 to 5 years of age, I usually begin with 2000 units, and above 5 years, 3000 units, repeating or raising the dose in twelve to eighteen hours if no distinct improvement. In laryngeal diphtheria or intense toxic conditions there is practically no limit to dosage—fifteen, twenty, thirty or even fifty thousand units. The success of the treatment depends on giving enough to counteract the effect of the toxins present. When enough is given results will be apparent. I have never yet had to resort to the larger dosage—twenty to fifty thousand units—but would not hesitate to do so in a desperate case.

Again, in laryngeal diphtheria, an intubation set should always be at hand and, although in some cases intubation is an extremely difficult operation, where it is possible it offers many advantages over tracheotomy. With a rebellious child it is not well to attempt swabbing or spraying or other local treatment. If a tractable patient, mopping with equal parts of listerine and peroxide of hydrogen keeps the throat and mouth in better condition. I also use an ointment of boric acid, gr. xv., zinc oxide, gr. xv., to an ounce of white vaseline, introducing a plug as large as the nasal cavity will admit in each nostril every three or four hours while the child is lying on its back. The body heat dissolves the ointment and allows it to pass back into the post nasal passages, producing a sedative and antiseptic effect. A nourishing and stimulating diet is kept up throughout the course of the disease, and a tendency to weak heart is met with strychnia, eggnog—milk punch and whiskey or brandy. On account of the many accidents which follow diphtheria, such as cardiac paralysis, neuritis, etc., the patient should be kept in bed fifteen to twenty days after apparent recovery. The following case will, perhaps, serve to illustrate the necessity of the after-care of these patients: R. L.; aged 8; Afro-American; brought to my office for sore throat; August 24th examination revealed naso-pharyngeal diphtheria; pulse 100°; temperature normal; nasal passages completely occluded; gave anti-toxin, 3000 units at 9 a. m. Just twenty-four hours later was called to see the child on account of nasal hemorrhage; parents stated that a brown discharge had been passing from nasal passages all day; the slight bleeding evidently due to the separation of membrane in post-nares. At this examination temperature normal; pulse about 55, but fairly strong; gave strychnia, gr. 1/100 every four hours and whiskey eggnog and milk punch ad. lib.; dressed nasal passages with hydrogen peroxide, which soon checked hemorrhage, which was not alarming, and left cotton plugs saturated with the peroxide in both anterior nares. On morning following (August 26th) I found patient doing well except pulse rate, which was regular and of good volume, but was only 40. Administered 1500 units anti-toxin and increased strychnia to 1/60 gr. every four hours. At my visit on morning of 27th found child up walking around the room in face of my instructions to the contrary and clamoring for something to eat. Pharynx, fauces and nose clean and clear; pulse 65, regular and of good volume. This was the last time I ever saw the patient alive. At 4:30 on afternoon of the 30th, in passing the house, I called unexpectedly to the family, and found the father of the child some fifty yards from the house with the child bare-headed in his arms, dead. It was a history of continued improvement—child playing, good appetite, a hot afternoon, thermometer registering 98° in the shade, no breeze, took child out to stool; the heat shock overcame the weak heart. This is the only case in my experience in which anti-toxin seemingly produced any result on the circulation except that of indirect stimulation.

Phophylactic: It is my invariable rule to inject antitoxin ranging in dose from 100 units in infants to 500 in children over 7 years of age who have been exposed. I have never had a failure to prevent the disease.

My experience leads me to believe that any case of diphtheria where the patient has eighteen hours to live may be saved under proper dosage and with the aid of intubation if necessary. I would except cardiac failure and broncho-pneumonia. The first may be largely obviated by proper nursing and cardiac stimulants with a weak heart. Much may be done to prevent broncho-pneumonia by keeping mouth, throat and nose clean. I prefer Stearns's antitoxin. I first used it because of its great convenience, and continue to use it because of its unfailing reliability. While there are many excellent serums on the market, I have not thought it wise to experiment with others.

